# Image Defogging Framework Using Segmentation and the Dark Channel Prior

**DOI:** 10.3390/e23030285

**Published:** 2021-02-26

**Authors:** Sabiha Anan, Mohammad Ibrahim Khan, Mir Md Saki Kowsar, Kaushik Deb, Pranab Kumar Dhar, Takeshi Koshiba

**Affiliations:** 1 Department of Computer Science and Engineering, Chittagong University of Engineering and Technology, Chattogram 4349, Bangladesh; sabiha.anan@cuet.ac.bd (S.A.); muhammad.ikhancuet@gmail.com (M.I.K.); sakikowsar@cuet.ac.bd (M.M.S.K.); pranabdhar81@cuet.ac.bd (P.K.D.); 2 Faculty of Education and Integrated Arts and Sciences, Waseda University, 1-6-1 Nishiwaseda, Shinjuku-ku, Tokyo 169-8050, Japan; tkoshiba@waseda.jp

**Keywords:** dark channel prior, floodfill algorithm, image blending, image enhancement, segmentation

## Abstract

Foggy images suffer from low contrast and poor visibility problem along with little color information of the scene. It is imperative to remove fog from images as a pre-processing step in computer vision. The Dark Channel Prior (DCP) technique is a very promising defogging technique due to excellent restoring results for images containing no homogeneous region. However, having a large homogeneous region such as sky region, the restored images suffer from color distortion and block effects. Thus, to overcome the limitation of DCP method, we introduce a framework which is based on sky and non-sky region segmentation and restoring sky and non-sky parts separately. Here, isolation of the sky and non-sky part is done by using a binary mask formulated by floodfill algorithm. The foggy sky part is restored by using Contrast Limited Adaptive Histogram Equalization (CLAHE) and non-sky part by modified DCP. The restored parts are blended together for the resultant image. The proposed method is evaluated using both synthetic and real world foggy images against state of the art techniques. The experimental result shows that our proposed method provides better entropy value than other stated techniques along with have better natural visual effects while consuming much lower processing time.

## 1. Introduction

During bad weather, the presence of fog in the atmosphere reduces perception of visibility. In some cases, images suffer severe degradation and contrast loss. Consequently, performance of different computer vision algorithms gets lowered due to the lack of quality images. Even the presence of fog disturbs the original scene structure of images. Therefore, defogging images has become a pivotal pre-processing step in the arena of computer vision.

To maintain high ratio of precision in the performance of computer vision algorithms, many fog removing methods have been introduced over the years. Some algorithms work on small features which are not defined clearly. A few algorithms require multiple images of the exact scene in order to estimate the depth information such as a polarization-based method [1,2]. Since multiple image-based methods are not effective, single image fog removal methods have become more popular. This can be done in two different ways: the techniques based on image enhancement and other on image restoration.

Image enhancement-based techniques work with improving image contrast [3]. The enhancement-based approaches include Histogram equalization [4], Retinex theory [5], Homomorphic filtering [6], Wavelet based scheme [7], etc. Most of these approaches have problems with maintaining color fidelity. In contrast, image restoration-based methods can provide natural defogging effects. However, different methods that are being used either do not give accurate results [8] or lead to halo effects in the final image [9]. Among the restoration techniques, the method proposed by He et al. [10] popularly known as Dark Channel Prior (DCP) technique was later studied for its simple principle and excellent effects. Still, this method is not effective for images which contain homogeneous regions like sky. The resultant image suffers from serious color distortion in the sky region.

Image segmentation plays a vital role in image processing. It is an important preprocessing step in computer vision. Images are segmented into multiple patches or segments and necessary tasks are performed on those segments. Later those segments are combined through image fusion. Various segmentation technique of images are proposed over the years. [11] proposed a remote sensing image fusion technique through compressive sensing-based method. To do this, a dictionary is constructed trained with patches across different image scales. Thus, it is called multiscale dictionary. Then an iterative minimization method is designed to seek the sparse coefficient vectors. This algorithm aims to use captured low resolution multispectral data to estimate missing high resolution multispectral data. However, this approach has very high computational complexity. A novel unsupervised segmentation algorithm based on Markov random field is proposed in [12]. Here, the segmentation algorithm uses class-driven vector data quantization and clustering. Based on these resultant clusters, the likelihoods are estimated. Though this method appears to be promising for peri-urban areas, application of the system in purely urban scenes would be a challenging job. In [13], an image defogging technique based on sky region segmentation is presented. In this paper, at first the foggy image is segmented into a rough binary image using Otsu method [14]. This rough binary image is refined using guided filtering. After that a modified DCP technique is used for image defogging so that the color distortion in sky region can be removed. Though color distortion and halo effects in sky region due to DCP are eliminated, however, this method cannot preserve the original color of the sky region.

To eliminate this issue regarding the distortion of sky region while using DCP, we proposed a method based on partitioning the sky portion and restoring the non-sky and sky part individually. As a result, the block effects caused by DCP can be removed. The sky region will be smoother and without any distortion. The proposed method restores the foggy image in an efficient way and requires much less computational time as compared to other techniques. It restores the original color of the initial foggy image. This method does not require any prior knowledge about the feature of each isolated image, thus can be used to defog images having different characteristics. Furthermore, the restored image is enhanced to remove the problem of over saturation and gives a more pleasing look. The key contributions of our proposed framework are as follows:

1. An efficient and fast sky part segmentation process from the foggy image is proposed.

2. The limitation of DCP technique is overcome and the original color of the sky region is preserved in the restored image.

3. An effective defogging approach is presented that consumes much less computational time.

4. The method does not require any prior knowledge about individual foggy image.

5. Our method is tested against both synthetic and natural images from RESIDE dataset [15], also against real world images. The experimental result shows that our approach outperforms the recent state-of-the-art techniques.

The rest of the paper is presented in the following sections. In Section 2 related works concerning defogging algorithms are provided. The conceptual background of the related topics are introduced in Section 3. Proposed methodology is covered in Section 4. Section 5 contains an analysis of the experimental results. Finally, we conclude this paper in Section 6.

## 2. Related Work

It is imperative to achieve accuracy in visibility of images for computer vision algorithms. The main reason behind invisibility during bad weather is attenuation and airlight. Hence estimation of depth is measured by using multiple images [16]. The methods based on depth information requires inputs from user or known 3D model [16]. Therefore, defogging the images required multiple images in early days. For instance, Polarization-based methods remove the haze effect by taking two or more images with different degrees of polarization [2]. However, multiple images of same scene cannot be obtained easily, that makes this method a not very effective one. Thus, the concentration is made on single image defogging techniques instead of multiple image defogging methods. At present, processing methods for foggy images mainly include image enhancement-based approaches and image restoration-based approaches. Here we introduce briefly these approaches for defogging single image.

### 2.1. Enhancement-Based Defogging Approaches

These methods simply improve image visual effects through contrast enhancement techniques. Image enhancement is a general approach to get enhanced quality images concerning human visual observation. In [4], histogram equalization can be used to three color channels separately in case of colored images. However, this technique is susceptible to change in hue and does not maintain color fidelity fully. Retinex theory [5] uses color constancy property. This characteristic confirms that the perceived color of objects remains same under altering light. Although the retinex algorithms are based on human visual perception, they use a localized version of this principle. However, human color perception is way more complex than that of retinex algorithm. Moreover Homomorphic filtering [6] is mainly used to increase contrast and normalize the brightness across the image. It is also used to remove multiplicative noise that ensures good dynamic range compression. The main disadvantage of the technique is that it does not produce proper color rendition. It is because the technique is used to improve the appearance of grayscale image. The method is not capable of enhancing the RGB image optimally which might cause image distortion. Thus, the stated algorithms do not conserve the color fidelity. In [17] the color fidelity is maintained by correcting the contrast loss. However, it does not provide much enhancement in visibility. These above mentioned techniques enhance the visual effects rather than actually defog the image.

### 2.2. Restoration-Based Defogging Approaches

Image restoration-based methods might obtain natural defogging effects. Tan’s method [18] restores an image contrast to its maximum extent; it produces oversaturated images because this method assumes that haze free images should have higher contrast than hazy images. The method involving independent component analysis and Markov Random Field is proposed by Fattal [8]. It recovers color image, though requires much time and exact color information of the scene. Tarel and Hautiere [9] have calculated atmospheric veil function with the help of median filtering and further used color tone mapping to have defogged image. The method however, produces images containing halo effects. A dehazing algorithm is proposed by Zhang et al. [19]; it depends on assumption of luminance variation. It also uses an edge preserving filter for sharpening the edges. However, the transmission map created by this technique is not correct. He et al. [10] introduces a Dark Channel Prior scheme that uses minimum filtering and optimization of the transmission map for achieving a good resultant image. Additionally, adaptive weiner filter [20] is used for defogging images. The method is less time consuming and comparable to dark channel prior to some extent. It is to note that mere filtering used by the method is not sufficient to remove fog from an image. For removing the haze a multiscale fusion method is used by Wang et al. [21]. Here, the technique, based on the existing dark channel prior, uses two atmospheric veils with different scales from the hazy image. The scene depth property is preserved through adaptive local similarity based wavelet fusion. However, this method is not practical as it cannot properly remove haze from the non homogeneous region and unnecessary textures in the scene depth exists. Presently, Artificial Intelligence (AI) based methods are also being used for defogging images [22,23,24,25,26,27] and deliver some promising results. However, these methods have higher computational complexity than traditional statistical based defogging approaches. Method described in [28] ensures a relatively fast convergence of training model with a homogeneous model generalization. However, the technique requires the original ground truth images of the foggy images, which are not available at all times. Therefore, it cannot be applied on real world images without prior knowledge. In [29], three scales convolutional neural network to predict transmission map is used. Then, fog free images are recovered using the atmospheric scattering model. It takes a lot of time to train the model as no prior assumptions are available. An end-to-end encoder decoder training model is used in [30] to get high quality dehazed image. Though it can remove haze efficiently, it has a very low processing speed, which makes it unsuitable for real time use.

Removing clouds from remote sensing images is a much-related task to image defogging. Several algorithms have been proposed for removing clouds from remote sensed images over the years. In [31], an algorithm depending on multitemporal dictionary learning is proposed for recovering quantitative data, which are contaminated by thick clouds and shadows. In this paper, two multitemporal learning algorithms such as K- Singular Value Decomposition (KSVD) and Bayesian methods are suggested. Both the methods are adaptively optimized for making better use of temporal correlations and offer very good output images. However, between these two, the Bayesian method achieves a more effective result than KSVD. KSVD gets better results when less data is used. For removing thick clouds, the Bayesian method is the better option to choose. Likewise, in [32] the authors proposed a network that can remove clouds and can generate visible light images from multispectral images. This was achieved by extending the input channels of conditional Generative Adversarial Networks (cGANs) to be compatible with multispectral images. However, this method can only remove thin clouds and used simulated clouds with Perlin noise for training and testing the model, which is quite different from the real cloud. Another approach of cloud removal from remote sensing images is presented in [33]. In this method, a non-negative matrix factorization and error correction method are used to recover cloud-free images. This method does not require cloud detection as a preprocessing step. However, the method needs cloud free reference images of the corresponding cloud contaminated satellite images.

Among the methods mentioned above, the DCP technique (He et al. [10]) draws significant attention for its simple principle and excellent effects. However, the DCP technique has some limitations, such as higher processing time as it uses soft matting for transmission map refinement and serious color distortion and noise in the sky region as the DCP assumption does not work perfectly on sky region. Here the transmission map obtained using DCP is improved further through bilateral filtering [34], WLS (Weighted Least Square) edge preserving smoothing [35], guided filtering [36]. Nevertheless, the distortion in sky region prevails. Therefore, the concept of sky area segmentation comes to the fore.

In our proposed method, the foggy image is divided into two parts sky and non-sky part; both the parts are restored separately. The non-sky region is restored through DCP technique while sky part is enhanced by Contrast Limited Adaptive Histogram Equalization (CLAHE) method. Therefore, the said problem of DCP technique can be eliminated. Moreover, the resultant images obtained from the proposed method are evaluated through different image performance evaluation metrics; such as Structural Similarity Index Measure (SSIM), Peak Signal-to-Noise Ratio (PSNR), Natural Image Quality Evaluator (NIQE), Contrast measurement and entropy calculation. The proposed method is compared with different state-of-the-art techniques. The experimental result shows that the images obtained from this method gives better defogged images as well as requires low processing time.

## 3. Background

### 3.1. Atmospheric Scattering Model

Airlight and attenuation are the main causes for image invisibility. Depending on this, the fog model can be illustrated as [10]:

(1) I(x)=J(x)t(x)+A(1−t(x))

where I(x) is foggy image intensity, J(x) stands for the intensity of defogged image, t(x) is transmission map and *A* is the global atmospheric light.

Based on (1), scene radiance of defogged image can be derived as [10]:

(2) $$J\left(x\right)=\frac{I(x)-A}{t(x)}+A$$

### 3.2. Dark Channel Prior

The dark channel prior method is primarily dependent on the nature of fog free images. Generally the area which are not covered by the sky contains some pixels having very low intensity, nearly zero in atleast one color channel [10].The equation can be written as follows [10]:

(3) $$J^{dark}\left(x\right)=\min_{c\epsilon r,g,b}\left(\min_{y\epsilon \Omega (x)}\left(J^{c}\left(y\right)\right)\right)$$

where $J^{c}$ is the color channel of *J* and Ω(x) is the local fragment centered at *x*.

For fog free image, the intensity of $J^{dark}$ tends to be zero [10].

(4) $$J^{dark}\left(x\right)=\min_{c\epsilon r,g,b}\left(\min_{y\epsilon \Omega (x)}\left(J^{c}\left(y\right)\right)\right)\to 0$$

The above statistical knowledge is called dark channel prior [10].

### 3.3. Transmission Map

To calculate estimated transmission map of foggy image, He et al. [10] performed double min operation on the fog model (1) among three color channels separately. Therefore, it can be written as [10]:

(5) $$\min_{c}\left(\min_{y\epsilon \Omega (x)}\left(\frac{I^{c}\left(y\right)}{A^{c}}\right)\right)=\bar{t}\left(x\right)\min_{c}\left(\min_{y\epsilon \Omega (x)}\left(\frac{J^{c}\left(y\right)}{A^{c}}\right)\right)+\left(1-\bar{t}\left(x\right)\right)$$

where $\bar{t}\left(x\right)$ is the estimated transmission map, $I^{c}\left(y\right)$, $J^{c}\left(y\right)$, $A^{c}$ are the *c*-th color component of input foggy image, defogged image and atmospheric light respectively.

From (4) and as $A^{c}$ is a positive constant, this leads to [10]:

(6) $$\min_{c}\left(\min_{y\epsilon \Omega (x)}\left(\frac{J^{c}\left(y\right)}{A^{c}}\right)\right)=0$$

Substituting (6) in (5), we get [10],

(7) $$\bar{t}\left(x\right)=1-\min_{c}\left(\min_{y\epsilon \Omega (x)}\left(\frac{I^{c}\left(y\right)}{A^{c}}\right)\right)$$

To perceive depth of the scene, a fixed parameter ω(0<ω≤1) is inaugurated, otherwise the image will look unnatural. This is called aerial perspective [10].

(8) $$\bar{t}\left(x\right)=1-\omega \min_{c}\left(\min_{y\epsilon \Omega (x)}\left(\frac{I^{c}\left(y\right)}{A^{c}}\right)\right)$$

### 3.4. Color Distortion of Sky Region

Serious noise and color alterations occur in the sky portion when the image is restored by dark channel prior. Hence a dark channel prior algorithm is not applicable to white regions such as sky [34]. It is because that the intensity value of the dark channel of sky region is always greater than zero [34]. Thus, the obtained transmission map will have values smaller than the real values for bright regions [37]. Therefore, the scene radiance of (2) is equivalent to [37],

(9) $$J\left(x\right)=I\left(x\right)+\left(\frac{1}{t(x)}-1\right)\left(I\left(x\right)-A\right)$$

**Figure 1:**
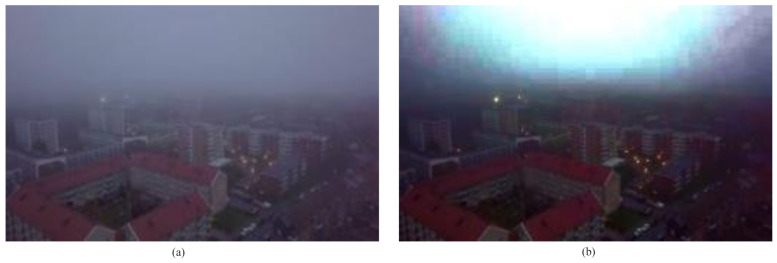
Image defogging using DCP (Dark Channel Prior) method. (**a**) Input image, (**b**) Output image.

Thus, because of large amplification factor, noise might be amplified and the color of the restored sky region will be distorted. Figure 1b shows the example of color distortion in the defogged image.

## 4. Proposed Methodology

**Figure 2:**
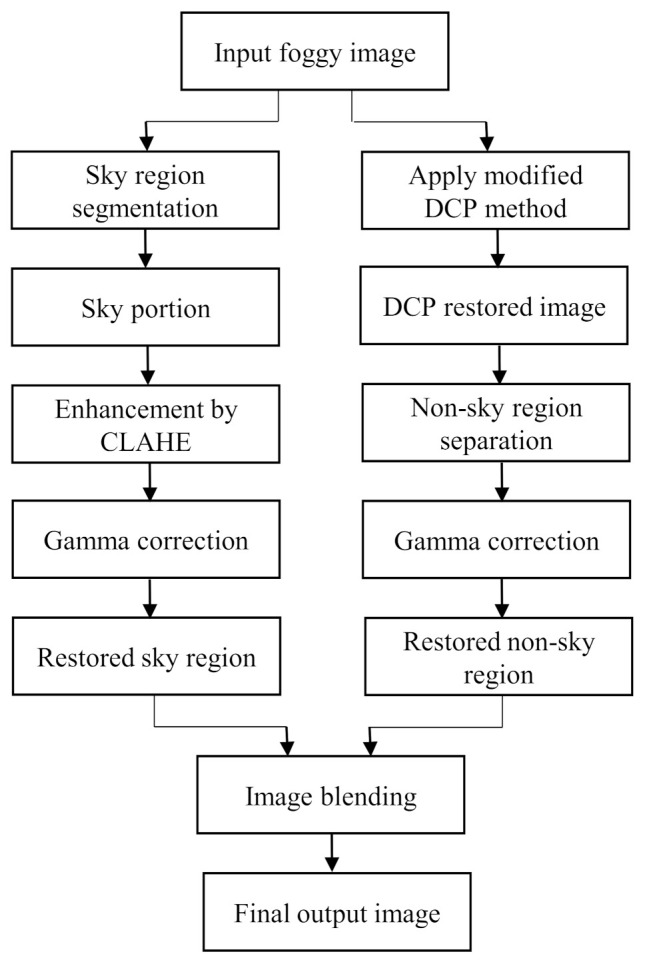
Flowchart of the proposed method.

The major focal point of this method is to surpass the limitation of the dark channel prior algorithm within a lower computational time while defogging a foggy image. Therefore, a method was proposed where sky and non-sky part are restored separately. As a result, the sky part is restored without having its color distorted. The original color of the sky can be preserved through this approach. Then the restored image is enhanced to have a good visual perception. The flowchart of the proposed framework is shown in Figure 2.

### 4.1. Sky Part Segmentation

**Figure 3:**
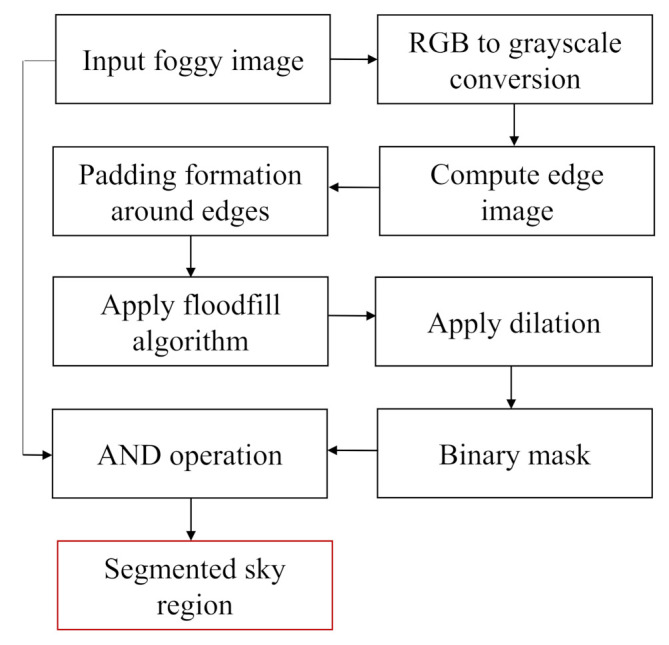
Flowchart of the sky segmentation process.

**Figure 4:**
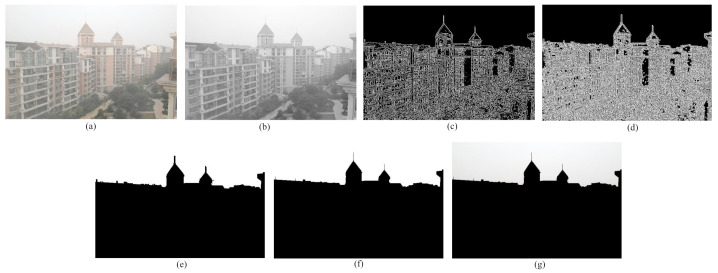
Sky region segmentation process. (**a**) Input foggy image, (**b**) grayscaled image, (**c**) edge image, (**d**) padding formation around edges, (**e**) application of floodfill algorithm, (**f**) dilated image, (**g**) segmented sky region using the binary mask and input image.

For foggy images containing large sky area, though the non-sky part’s color is comparatively vivid, in most cases the sky region looks gray and homogeneous. To separate the sky region, a separation method based on the floodfill algorithm is introduced. The flowchart for sky portion segmentation process is presented in Figure 3. The description of each step is given below:

**Step 1:** Input foggy image is converted into grayscale image as shown in Figure 4b.

**Step 2:** The edge image of the grayscale image is computed through canny edge detection algorithm [38] as depicted in Figure 4c.

**Step 3:** In most of the cases, the edge image contains discontinuities among the edges. Therefore, to precisely separate sky and non-sky part, the disruption among the broken edges are to be filled up. To do so, Adaptive Mean Thresholding is used on the edge image. This technique dynamically selects threshold value over an image. The threshold value is the mean of neighborhood area. The neighborhood area is a fixed size window around the pixel location [39]. As this method is used on an edge image, a padding of the size of window is formed around the edge pixels as shown in Figure 4d. Thus, the disruption can be eliminated and a well defined boundary between the sky and non-sky part can be obtained.

**Figure 5:**
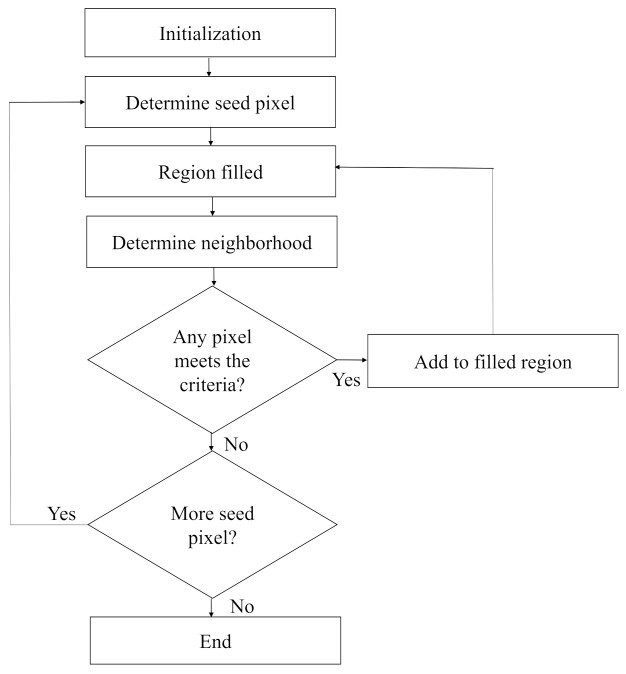
Work flow of floodfill algorithm for determining the sky region.

**Step 4:** To acquire the sky region, floodfill algorithm is used. In this paper it is considered that the sky region usually resides at the top part of the image. Each of the pixels in the first row is treated as the seed point. At the first seed point, if the pixel is black, it is replaced by white color and its four neighbors are pushed into the stack. Thereafter, the topmost pixel value is popped up and considered as the next seed point. The same procedure will be carried out for all the seed pixels until any of them is left. The working procedure of this algorithm is shown in a flowchart in Figure 5. The image obtained after the implementation of floodfill algorithm is a binary image where the sky part represents the foreground and the non-sky part represents the background which is presented in Figure 4e.

**Step 5:** To eliminate the unwanted discontinuities in the sky area and in the boundaries of the sky area, dilation is performed. Through the dilation process, pixels are added to the object boundaries. Finally, a binary mask is obtained where the white region represents the sky portion of the input foggy image. Figure 4f represents the acquired binary mask.

**Step 6:** The obtained binary mask is used to isolate the sky region from the non-sky part of the original image. To do this, an AND operation is performed between the binary mask and the input foggy image. The resultant sky region is displayed in Figure 4g.

### 4.2. Restoration of Sky Part

**Figure 6:**
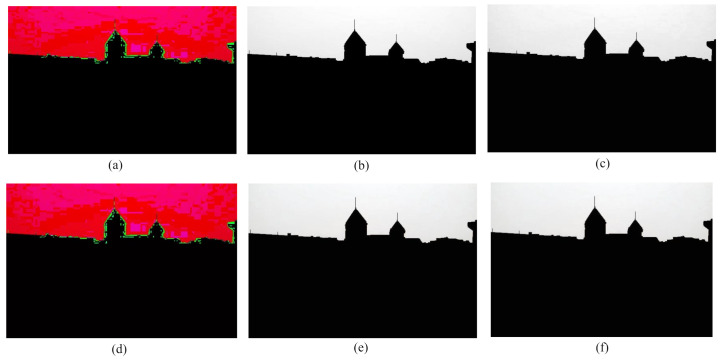
Sky region restoration process. (**a**) HSI image of foggy sky region, (**b**) intensity (I) component of HSI image, (**c**) application of CLAHE on intensity (I), (**d**) merge H, S, and new I, (**e**) defogged sky region in RGB color space, (**f**) restored sky region after gamma correction.

The color information of an homogeneous region is not maintained in DCP method. Therefore, to restore the sky region within a lower computational time, we chose to defog it with one of the enhancement techniques [40]. The steps for sky region defogging are as follows:

**Step 1:** Transformation of the sky region of foggy image, shown in Figure 4g from RGB to HSI color space as appeared in Figure 6a.

**Step 2:** Splitting the H (Hue), S (Saturation) and I (Intensity) components of the obtained image. The intensity component image is presented in Figure 6b.

**Step 3:** Application of CLAHE on the intensity (I) component only. [It is due to the fact that hue and saturation of a foggy image and its defogged counterpart should remain same [41]. The obtained image is shown in Figure 6c.

**Step 4:** Merging H, S and the new I. The resultant HSI image is displayed in Figure 6d.

**Step 5:** Conversion from HSI color space to RGB color space. Figure 6e depicts the defogged sky area.

**Step 6:** To give the restored image a clearer look, further enhancement of the image is necessary. Therefore, the gamma correction method is used to enhance the image further. Gamma correction can be expressed by the power law expression as follows [42]:

(10) $$I_{out}=cI_{in}^{\gamma }$$

where the output image $I_{out}$ is obtained from the input image $I_{in}$ which is raised to the power γ and multiplied by a constant, *c*. The consequent restored sky region image is shown in Figure 6f.

### 4.3. Restoration of Non-Sky Part

To defog the non-sky region of an image, we will use dark channel prior [10] technique for its excellent defogging results. However, the traditional DCP method [10] uses soft matting for transmission map refinement which consumes high computational time. Therefore, to lower the computational cost we used Guided Filtering technique [36] instead of soft matting for refinement of the transmission map. The restoration of non-sky part is described below:

**Step 1:** Estimation of the airlight A. For this, 0.1% of the peak pixels in the fog degraded image is taken and the highest intensity among those pixels is considered as the atmospheric light.

**Figure 7:**
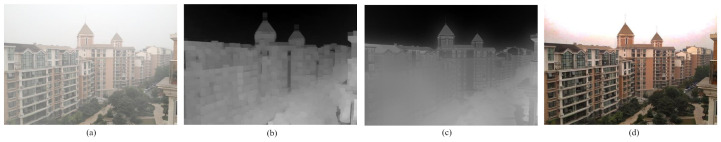
Dark channel prior process. (**a**) Input foggy image, (**b**) estimated transmission map $\bar{t}\left(x\right)$, (**c**) refined transmission map t(x), (**d**) resultant DCP image.

**Step 2:** The estimated transmission map $\bar{t}\left(x\right)$ for the foggy image is found out through (8). Figure 7b depicts the estimated transmission map of input foggy image Figure 7a.

**Step 3:** The restored image might suffer from block effects while using the estimated transmission map that is obtained. Thus, a guided filtering technique is used to immaculate the transmission map. For guided filtering, we used the input image as the guidance image and the estimated transmission map as the image to be filtered [36]. The refined transmission map t(x) is shown in Figure 7c.

**Step 4:** The scene radiance of the DCP restored image can be recovered using (2). The transmission is confined to a lower bound $t_{0}$. Thus, (2) can be written as

(11) $$J\left(x\right)=\frac{I(x)-A}{max(t\left(x\right),t_{0})}+A$$

The resultant DCP restored image is shown in Figure 7d.

### 4.4. Separation and Enhancement of Non-Sky Part

**Figure 8:**
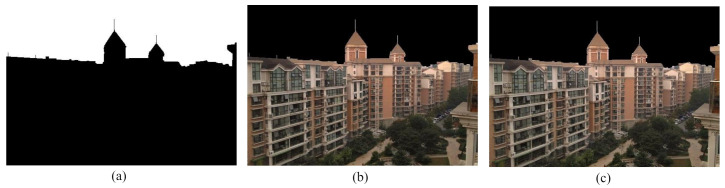
Non-sky region segmentation and enhancement process. (**a**) Inverted binary mask, (**b**) segmented non-sky region from the DCP restored image, (**c**) restored non-sky region after gamma correction.

The invert of binary mask discussed in Section 4.1 has the non-sky part as foreground and the sky part as background. The inverted binary mask is shown in Figure 8a. This mask is used to detach the non-sky part and sky part from the DCP restored image following the same procedure as mentioned in Section 4.1. Here, the AND operation is performed between the inverted binary mask and the DCP restored image. The obtained non-sky region is exhibited in Figure 8b.

The image obtained after performing the dark channel prior method usually have high contrast, i.e., the images are extremely dark in dark areas and extremely white in white areas. Therefore, the objects in the images cannot be distinguished clearly. To unravel this problem, we used gamma correction technique [42] which follows the equation given in (10). The restored non-sky part is shown in Figure 8c.

### 4.5. Image Blending

**Figure 9:**
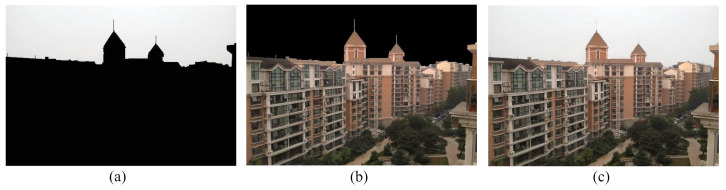
Image blending process. (**a**) Restored sky region, (**b**) restored non-sky region, (**c**) final restored image.

The restored sky and non-sky part are blended together to obtain the resultant image. The equation for alpha blending is given below:

(12) $$I_{blend}\left(x\right)=\alpha I_{non-sky}+\left(1-\alpha \right)I_{sky}$$

where $I_{non-sky}$ and $I_{sky}$ are restored non-sky and sky area respectively. α is called blending ratio. It designates how the output image is influenced by each input image. It can either be a constant factor for all of the pixels in the image or it can be determined separately for every pixel with the help of a mask. In our proposed framework, the invert of the binary mask formulated previously by floodfill algorithm shown in Figure 8a is used as the alpha mask. Hence, the final output image is displayed in Figure 9c.

## 5. Experimental Result Analysis

### 5.1. Comparison with DCP Image

**Figure 10:**
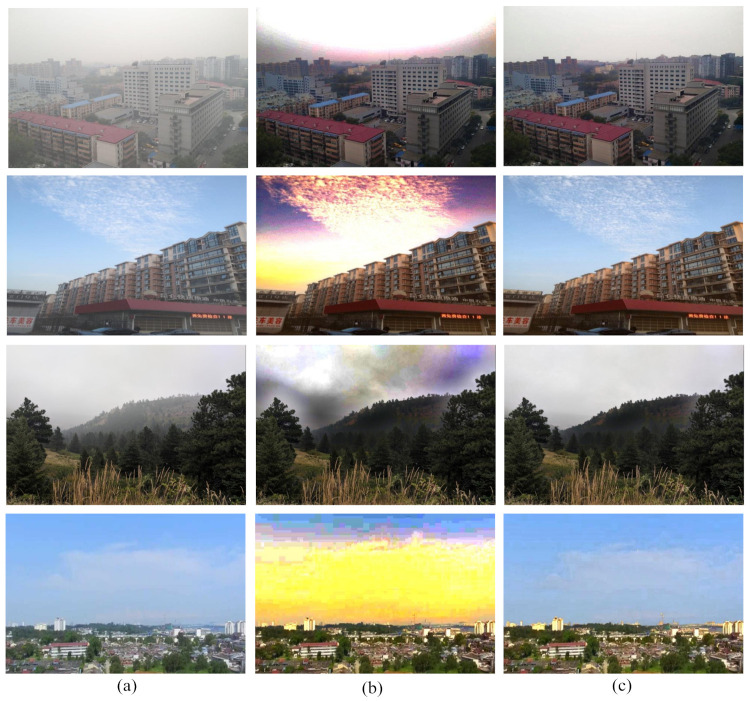
Comparison among (**a**) Foggy images, (**b**) images after applying DCP method, and (**c**) images after applying proposed method.

Some foggy images and their corresponding defogged images using our proposed method are exhibited in Figure 10. The input images are evaluated with both the DCP and our proposed method through visual perspective. The proposed technique overcomes the color distortion problem of the sky region. This comparison represents how our proposed method overcomes the color distortion problem of the sky region inherited from the dark channel prior technique.

### 5.2. Comparison with Other Techniques

In this section, the benefits of our proposed method are evaluated in terms of computational complexity with respect to images of different sizes. Table 1 represents the comparative analysis of computational complexity with the G. Wang’s method [43], W. Wang’s method [37], two methods proposed by A. Sabir [44], S. Salazar-Colores’s method [45] and our proposed method. Our proposed method requires much less processing time than other stated techniques under similar software and hardware conditions. It is to be mentioned here that G.Wang’s technique [43] requires less time; the segmentation between the non-sky and sky part, however, is not precise. Thus, the border between the sky and non-sky part is clearly visible. Furthermore the method is only suitable with images having large sky region. In contrast, W. Wang’s method [37] uses quadtree decomposition to automatically select the seed point. It can effectively segment sky and non-sky part by using a region growing algorithm. The main disadvantage of this method is that it has a very high computational complexity. Additionally, the technique uses customized coefficient in the region growing criteria which varies from image to image. Thus, the value should be changed according to the image to get the best result. The first method proposed by A. Sabir [44] is a modified dark channel prior for image defogging. It is a slight modification of the original dark channel prior technique. Therefore, the color distortion of sky region remains to some extent. Besides it calculates the fog density of every image which increases the computational complexity of the method. The second method of A. Sabir is based on the segmentation of the sky region from the foggy image. However, the main drawback of this technique is that it is not fully automated. A semi-automatic approach is used to separate the foggy sky and non-sky region. S. Salazar-Colores’s method [45] gives a better defogged result, though the computational complexity increases with the size of the image.

Our proposed method has been executed in an experimental platform built in Python and the operating system is Windows 10. The hardware used in this experiment is a Dell laptop with Intel^®^ Core i3-5005U CPU @ 2.00 GHz and 4G RAM.

#### 5.2.1. **Full Reference Image Quality Evaluation**

**Figure 11:**
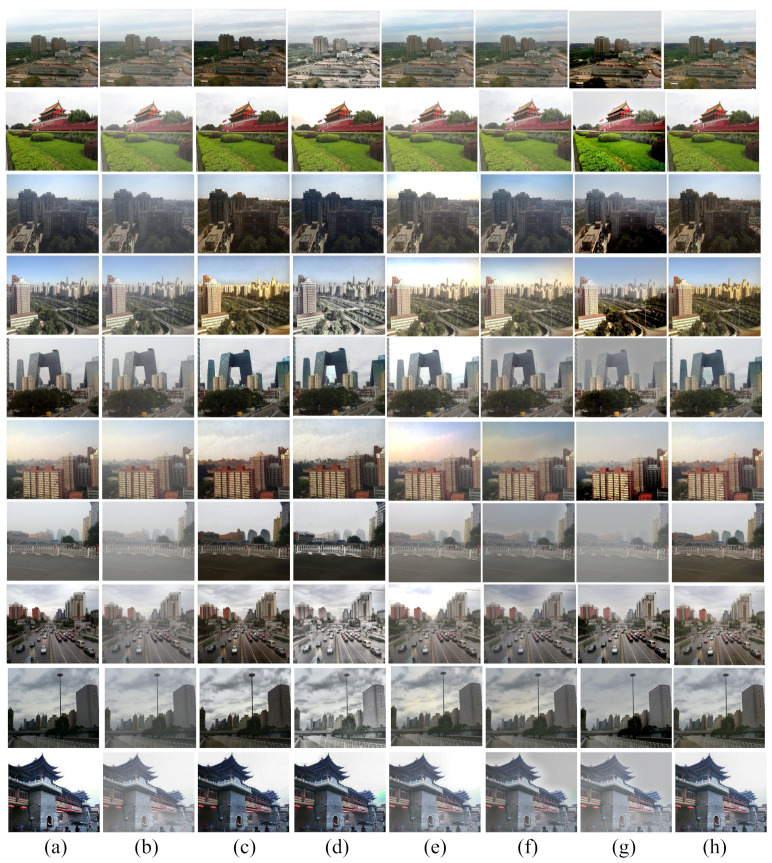
Comparison among different defogging algorithms using synthetically generated hazy dataset (RESIDE) [15]. (**a**) Ground truth images, (**b**) corresponding foggy images, (**c**) G. Wang et al.’s method, (**d**) W. Wang et al.’s method, (**e**) A. Sabir et al.’s method 1, (**f**) A. Sabir et al.’s method 2, (**g**) S. Salazar-Colores et al.’s method, and (**h**) proposed method.

To evaluate the result of our proposed algorithm, full reference metrics such as structural similarity index metrics (SSIM) [46], and peak signal to noise ratio (PSNR) have been taken into consideration. Besides, some non-reference metrics including naturalness image quality evaluator (NIQE) [47], contrast comparison and entropy measurement are taken into account. Through these matrices, the proposed algorithm is to be compared with the recent state of the art techniques. Figure 11 shows the qualitative comparison among different defogging techniques with the proposed method on images from RESIDE dataset [15].

**Figure 12:**
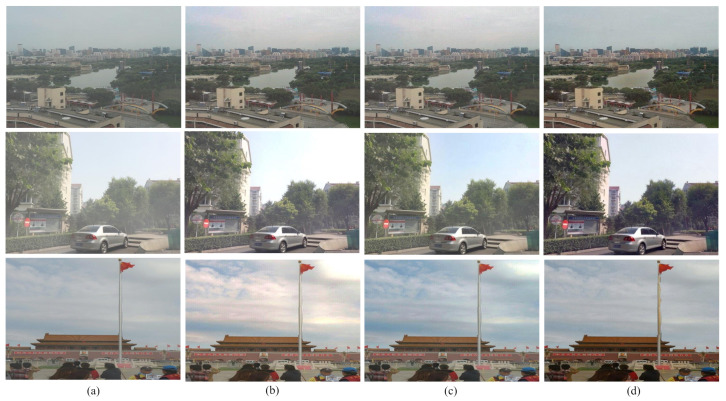
Comparison among (**a**) Foggy images, (**b**) images after applying Sabir et al.’s method 1, (**c**) images after applying Sabir et al.’s method 2, and (**d**) images after applying proposed method.

Figure 12 shows the comparison between the two proposed methods by Sabir et al. [44] and our proposed framework. Sabir et al.’s method 1 used a modified DCP technique for image defogging. Even if it gives better defogging result than the original DCP method, the color distortion in the sky region prevails. The second method proposed by Sabir et al. used segmentation to reduce the color distortion in the sky region. However, their segmentation-based defogging approach is semi-automatic. In other words, the separation of sky and non-sky parts is done manually. Besides, the original color and texture of the sky region in fog free image is not restored completely in this technique.

*SSIM* is a full reference based metric. Depending on contrast, brightness and structure, *SSIM* computes the similarity between the resultant image and the ground truth image. The range of *SSIM* value is between 0 and 1. The higher the *SSIM* value, the more the resultant image is similar to the ground truth image and is more appealing to the researchers. It is calculated by:

(13) $$SSIM=\frac{\left(2\mu_{xi}\mu_{yo}+k_{1}\right)\left(2\sigma_{xiyo}+k_{2}\right)}{\left(\mu_{xi}^{2}+\mu_{yo}^{2}+k_{1}\right)\left(\sigma_{xi}^{2}+\sigma_{yo}^{2}+k_{2}\right)}$$

where xi and yo are two common sized window, $\mu_{xi}$ and $\mu_{yo}$ are mean of the windows, $\sigma_{xi}$ and $\sigma_{yo}$ are their standard deviations, $k_{1}$ and $k_{2}$ are constants.

Mean squared error (*MSE*) is a full reference metric which is computed by:

(14) $$MSE=\frac{1}{mn}\sum_{a=0}^{m-1}\sum_{b=0}^{n-1}{\left[I_{in}\left(a,b\right)-I_{out}\left(a,b\right)\right]}^{2}$$

where $I_{in}$ and $I_{out}$ are input and resultant fog free image respectively, m×n is the size of the image. As *MSE* calculates the mean error between the two images, the less the error value, the better would be the result.

Peak Signal to Noise Ratio (*PSNR*) measures the quality of reconstruction of an image. The more the value of *PSNR*, the better the quality of the resultant image. *PSNR* value is calculated from *MSE* in the following way:

(15) $$PSNR=10\log_{10}\frac{MAX^{2}}{MSE}$$

where MAX represents the highest possible intensity value of an input image.

**Figure 13:**
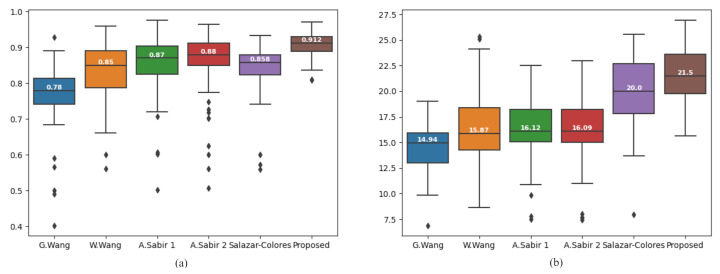
Box-plot comparison among state-of-the-art algorithm using 150 outdoor hazy images. (**a**) SSIM index, (**b**) PSNR value.

Table 2 and Table 3 presents the comparative results of the metrics, *SSIM*, and *PSNR* respectively computed over the ten images given in Figure 11. The results show that our proposed algorithm gives higher *SSIM*, *PSNR* values compared to the stated methods. We also computed *SSIM* index value, and *PSNR* values over 150 hazy images from RESIDE dataset to evaluate our proposed method against other methods. The results are presented in a box-plot implementation. The *SSIM* index box-plot, and *PSNR* box-plot implementations are shown in Figure 13a,b respectively.

#### 5.2.2. **No Reference Image Quality Evaluation**

**Figure 14:**
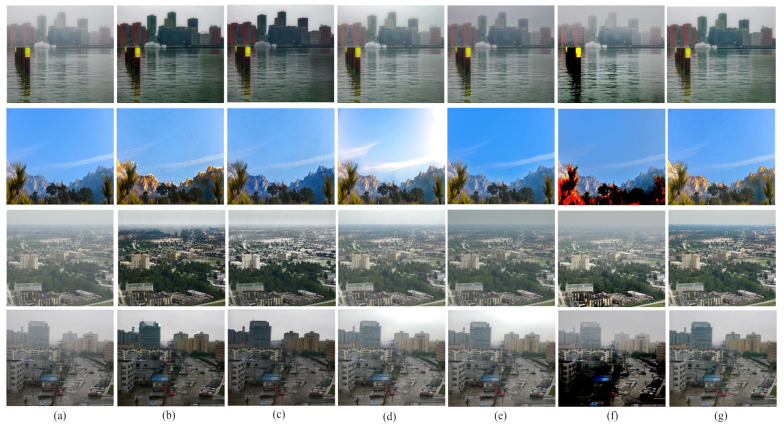
Comparison among different defogging algorithms using real world images. (**a**) foggy images, (**b**) G. Wang et al.’s method, (**c**) W. Wang et al.’s method, (**d**) A. Sabir et al.’s method 1, (**e**) A. Sabir et al.’s method 2, (**f**) S. Salazar-Colores et al.’s method, and (**g**) proposed method.

We evaluated our proposed method with real world images also. Figure 14 depicts the qualitative comparison among various techniques using some real world images. For real world images, the performance is evaluated with naturalness image quality evaluator (NIQE), contrast and entropy comparison. They are widely used in non reference image quality evaluation process. Table 4 shows the performance measure of the current state of the art methods with respect to NIQE for four real world images given in Figure 14. Here in NIQE, lower the value, the better. From the observed result, we see that the proposed method gives the lowest NIQE value. Therefore, it gives the best outcome in this aspect.

Contrast is defined as the distinction in luminance that makes the representation of the objects in an image distinguishable. For the comparison we used RMS (Root Mean Square) contrast. It is defined as the standard deviation of the pixel intensities. It is measured with the help of the following formula:

(16) $$\sqrt{\frac{1}{XY}\sum_{a=0}^{Y-1}\sum_{b=0}^{X-1}{\left(I_{ab}-\bar{I}\right)}^{2}}$$

where $\bar{I}$ is the mean intensity of all the pixel values in the image, image size is *X* by *Y* and $I_{ab}$ is the *a*-th *b*-th element of the image. Table 5 shows the comparative result of contrast comparison.

Entropy is defined as the measurement of information content in an image. It represents the intensity level which each pixel can adapt. It is used for the evaluation of image details; the higher the value of entropy, the more detailed the information are. For measuring the entropy of images, we used Shannon’s entropy as it measures the uncertainty of source of information [48]. Shannon’s entropy is defined as follows [48]:

(17) $$E\left(I\right)=-\sum_{k=0}^{L-1}p\left(k\right)\log_{2}\left(p\left(k\right)\right)$$

where E(I) is the entropy of image *I*, p(k) denotes the probability of occurance of value *k* in *I*, *L* indicates the number of gray levels. The entropy comparison among the recent state-of-the-art methods is depicted in Table 6.

In our proposed method, the DCP technique is used to restore the foggy images. It is based on a statistical approach and works pretty well unless there is no homogeneous sky region present in the image. The homogeneous sky region gets distorted, if the image is restored with DCP technique. Thus, we suggested to split the sky and non-sky portion of the image and restore them separately. The non-sky part is restored through DCP technique and sky part with the help of CLAHE. Since homogeneous sky region involves no edge, restoration through enhancement is a good choice. It requires less computational time. Moreover, the original color can be restored through CLAHE. Finally, the restored non-sky and sky portion are merged together. The resultant image is found distortion free. It is to be mentioned that no prior knowledge about the characteristics or behaviour of each image is required in our proposed technique. Thus, it provides extensive coverage of images. Notably, the images obtained by using He et al.’s method might suffer from oversaturation [17]. The Gamma correction technique is used to overcome the problem. It also adjusts the image based on human visual perception. Consequently, the obtained images in our proposed technique are more comprehensible, having defined edges with pleasant appearance.

## 6. Conclusions

In digital image processing, removing fog from foggy images is a primitive step. To achieve the objective, we performed segmentation of sky and non-sky part, then restored the sky and non-sky part separately. After amalgamation of both the restored parts, we found clearer visual perception of the foggy images. We evaluated our proposed framework with the five state of the art techniques. In all cases, both the synthetic and real world images have been used. The synthetic images are evaluated using SSIM index, and PSNR values and the real world images are assessed using NIQE, contrast as well as entropy measurement. Our framework displayed better result in both the cases. Moreover, it also takes much less processing time than other techniques under similar hardware and software environment. In case of heavily fogged images, the output in our proposed technique requires more precision. Therefore, there is a scope of further improvement.

## Figures and Tables

**Table 1:** Computational complexity comparison among different techniques.

Image Size	G. Wang et al.’s Method (s)	W. Wang et al.’s Method (s)	A. Sabir et al.’s Method 1 (s)	A. Sabir et al.’s Method 2 (s)	S. Salazar-Colores et al.’s Method (s)	Proposed Method (s)
320 × 180	0.67	3.55	2.55	3.07	3.06	**0.58**
600 × 400	2.29	5.66	5.35	6.66	11.76	**1.7**
640 × 480	2.67	8.14	7.68	8.53	19.9	**2.1**
800 × 600	2.8	10.45	12.19	13.64	21.38	**2.7**
620 × 950	3.85	11.52	14.29	15.26	37.88	**3.28**
1024 × 768	5.4	10.95	18.69	20.76	47.21	**4.7**
1280 × 860	6.67	18.45	26.51	28.88	63.91	**5.3**

**Table 2:** SSIM comparison among different methods over the 10 images from Figure 11.

Image no.	G. Wang et al.’s Method	W. Wang et al.’s Method	A. Sabir et al.’s Method 1	A. Sabir et al.’s Method 2	S. Salazar-Colores et al.’s Method	Proposed Method
1	0.896	0.793	0.945	0.946	0.853	**0.964**
2	0.835	0.926	0.836	0.838	0.818	**0.938**
3	0.861	0.901	0.952	**0.968**	0.868	0.965
4	0.908	0.824	0.938	0.941	0.821	**0.954**
5	0.902	0.900	0.862	0.865	0.855	**0.928**
6	0.814	0.926	**0.962**	0.960	0.854	0.954
7	0.801	0.811	0.960	0.955	0.873	**0.968**
8	0.903	0.759	0.856	0.872	0.900	**0.938**
9	0.868	0.834	0.849	0.848	0.876	**0.934**
10	0.904	0.939	0.811	0.809	0.725	**0.952**
Average	0.869	0.861	0.897	0.900	0.844	**0.949**

**Table 3:** PSNR comparison among different methods over the 10 images from Figure 11.

Image no.	G. Wang et al.’s Method	W. Wang et al.’s Method	A. Sabir et al.’s Method 1	A. Sabir et al.’s Method 2	S. Salazar-Colores et al.’s Method	Proposed Method
1	16.15	10.12	16.71	15.90	16.18	**22.87**
2	17.98	18.97	11.37	11.40	15.23	**22.29**
3	14.99	15.48	15.16	17.93	15.28	**23.12**
4	16.79	11.87	11.73	18.14	14.84	**20.69**
5	18.51	18.75	13.90	14.94	15.91	**20.12**
6	15.55	18.11	19.64	15.32	15.24	**21.28**
7	14.60	15.14	18.93	14.62	19.89	**22.12**
8	17.91	13.05	11.82	12.88	14.78	**21.27**
9	18.67	11.68	13.57	13.47	16.48	**21.80**
10	14.18	20.54	10.72	10.62	14.32	**20.59**
Average	16.53	18.43	14.35	14.52	15.82	**21.62**

**Table 4:** NIQE comparison among different methods over the four images from Figure 14.

Image no.	Foggy Image	G. Wang et al.’s Method	W. Wang et al.’s Method	A. Sabir et al.’s Method 1	A. Sabir et al.’s Method 2	S. Salazar-Colores et al.’s Method	Proposed Method
1	2.12	2.18	1.96	2.16	2.13	2.19	**1.93**
2	2.43	2.38	2.37	2.40	2.39	2.56	**2.36**
3	3.22	2.84	3.03	3.43	3.42	2.93	**2.44**
4	2.21	2.23	2.03	2.16	1.96	2.15	**1.86**

**Table 5:** Contrast comparison among different methods over the four images from Figure 14.

Image no.	Foggy Image	G. Wang et al.’s Method	W. Wang et al.’s Method	A. Sabir et al.’s Method 1	A. Sabir et al.’s Method 2	S. Salazar-Colores et al.’s Method	Proposed Method
1	44.93	61.36	61.60	48.28	37.08	63.61	**68.64**
2	73.27	58.96	71.63	73.93	73.38	**88.90**	73.75
3	43.21	74.74	67.37	54.24	39.22	68.65	**76.12**
4	54.70	69.47	67.74	65.59	69.15	75.69	**77.23**

**Table 6:** Entropy comparison among different methods over the four images from Figure 14.

Image no.	Foggy Image	G. Wang et al.’s Method	W. Wang et al.’s Method	A. Sabir et al.’s Method 1	A. Sabir et al.’s Method 2	S. Salazar-Colores et al.’s Method	Proposed Method
1	7.19	7.27	7.36	7.46	7.06	7.11	**7.54**
2	7.28	7.03	7.41	6.92	7.15	6.66	**7.53**
3	7.12	7.18	7.60	7.47	7.20	7.43	**7.80**
4	7.29	7.25	7.06	7.48	7.17	6.31	**7.51**

## Data Availability

The authors have used publicly archived hazy image dataset named RESIDE for validating the experiment. The dataset is available at https://sites.google.com/view/reside-dehaze-datasets/reside-v0 (accessed on 24 February 2021).
